# Management of Lung Cancer-Associated Malignant Pericardial Effusion with Intrapericardial Administration of Carboplatin: A Retrospective Study

**DOI:** 10.3390/curroncol29010015

**Published:** 2021-12-30

**Authors:** Hisao Imai, Kyoichi Kaira, Ken Masubuchi, Koichi Minato

**Affiliations:** 1 Division of Respiratory Medicine, Gunma Prefectural Cancer Center, Ota 373-8550, Japan; kmasubuchi@gunma-cc.jp (K.M.); kminato@gunma-cc.jp (K.M.); 2 Department of Respiratory Medicine, Comprehensive Cancer Center, International Medical Center, Saitama Medical University, Hidaka 350-0495, Japan; kkaira1970@yahoo.co.jp

**Keywords:** acute pericarditis, catheter drainage, intrapericardial carboplatin, lung cancer, malignant pericardial effusion

## Abstract

It has been reported that 5.1–7.0% of acute pericarditis are carcinomatous pericarditis. Malignant pericardial effusion (MPE) can progress to cardiac tamponade, which is a life-threatening condition. The effectiveness and feasibility of intrapericardial instillation of carboplatin (CBDCA; 150 mg/body) have never been evaluated in patients with lung cancer, which is the most common cause of MPE. Therefore, we evaluated the effectiveness and feasibility of intrapericardial administration of CBDCA following catheter drainage in patients with lung cancer-associated MPE. In this retrospective study, 21 patients with symptomatic lung cancer-associated MPE, who were administered intrapericardial CBDCA (150 mg/body) at Gunma Prefectural Cancer Center between January 2005 and March 2018, were included. The patients’ characteristics, response to treatment, and toxicity incidence were evaluated. Thirty days after the intrapericardial administration of CBDCA, MPE was controlled in 66.7% of the cases. The median survival period from the day of administration until death or last follow-up was 71 days (range: 10–2435 days). Grade 1–2 pain, nausea, fever, and neutropenia were noted after intrapericardial CBDCA administration. No treatment-related deaths were noted in the current study. Intrapericardial administration of CBDCA (150 mg/body) did not cause serious toxicity, and patients exhibited promising responses to lung cancer-associated MPE. Prospective studies using larger sample sizes are needed to explore the efficacy and safety of this treatment for managing lung cancer-associated MPE.

## 1. Introduction

It has been reported that 5.1–7.0% of acute pericarditis are carcinomatous pericarditis [1]. Malignant pericardial effusion (MPE) can progress to cardiac tamponade, a potentially life-threatening condition [2,3]. Lung cancer is a major cause of cancer-related deaths worldwide [4], and is the most common cause of MPE [5]. Patients with lung cancer-associated MPE show a poor prognosis, with a median survival time of 3 months or less [6,7]. Drainage of the pericardial fluid is recommended for long-term pericardial fluid control since there is a high chance of fluid re-accumulation after a single puncture. In a randomized controlled study evaluating the efficacy of bleomycin (BLM) in the management of MPE in 79 patients with non-small cell lung cancer (NSCLC) (JCOG9811), there was a trend toward a better survival in patients administered BLM (29% in the drainage alone group vs. 46% in the BLM group, *p* = 0.086). Although the primary endpoint (survival without pericardial effusion 2 months after drainage) did not differ significantly between the groups, the trend suggested a longer overall survival (OS) (median 79 days vs. 119 days) [8]. Various drugs have been evaluated to achieve pericardial adhesion in lung cancer. The pericardial fluid control rate at 30 days and median OS were reported to be 46–95% and 119–125 days, respectively, in the BLM group [8,9]; 75% and 80 days, respectively in the mitomycin C group [10]; and 80% and 69 days, respectively, in the carboplatin (CBDCA) group [11]. Moriya et al. reported ten cases where CBDCA 300 mg/body was administered intrapericardially for the management of carcinomatous pericarditis in patients with NSCLC; however, only a small number of patients was included in the study [11]. A previous study reported the efficacy of 150 mg intrapericardial CBDCA in the treatment of carcinomatous pericarditis in breast cancer [12]. According to the report, a response rate of 100% was achieved via this treatment, and no serious adverse events were observed. Although pericardiectomy is a treatment option for hemodynamic instability, no reported studies have prospectively evaluated the effect of pericardial effusion control. The decision should be based on the patient’s medical condition and the experience of physicians at the institution. Consequently, it is difficult to conduct randomized controlled trials due to the small number of subjects; therefore, there is currently insufficient evidence to determine a definitive treatment.

Several promising agents, such as immune checkpoint inhibitors and molecular targeting drugs, have been developed to improve the survival of cancer patients, in addition to improving palliative care. However, intrapericardial administration of therapeutic agents did not produce significant progress in patients with carcinomatous pericarditis. It remains unclear which agents are better at managing malignant effusions, since studies previously conducted have used small sample sizes. Therefore, we investigated the clinical effectiveness and feasibility of intrapericardial CBDCA (150 mg/body) administration after catheter drainage in patients with MPE associated with lung cancer.

## 2. Materials and Methods

### 2.1. Patients and Treatment Methods

We reviewed 21 consecutive medical charts of patients with lung cancer (NSCLC and small cell lung cancer (SCLC)) who had been administered intrapericardial CBDCA for the treatment of MPE at the Gunma Prefectural Cancer Center between January 2005 and March 2018. We retrospectively assessed the clinical effectiveness and adverse event profile of intrapericardial administration of fixed-dose CBDCA after catheter drainage in patients with MPE associated with lung cancer. The eligibility criteria were as follows: (i) histologically and/or cytologically proven NSCLC or SCLC; (ii) cytologically confirmed MPE causing large symptomatic pericardial effusion or cardiac tamponade. Lung tumors were pathohistologically classified according to the 2015 World Health Organization system. The patients’ charts were reviewed to collect data regarding the baseline characteristics and response to intrapericardial administration of fixed-dose CBDCA following catheter drainage. The study design was approved by the Institutional Review Board of the Gunma Prefectural Cancer Center. The need for informed consent was waived owing to the retrospective nature of the study. All patients were intrapericardially administered fixed-dose CBDCA, prior to pericardial adhesion. A drainage tube was inserted percutaneously into the pericardial space, under echocardiogram guidance. Following complete drainage of the effusion, a fixed dose of 150 mg CBDCA dissolved in 20 mL saline was administered into the pericardial space via the tube. Then, the latter was clamped and reopened after 2 h. When the drained effusion volume reached less than 50 mL/day, the drainage tube was withdrawn. In patients whom the drainage tube could not be withdrawn within 7 days of therapy, a second dose of CBDCA was administered. If MPE was controlled after pericardial adhesion, the patient was permitted to undergo subsequent treatment after consultation with the attending physician.

### 2.2. Evaluation of Response to Treatment and Adverse Events

The treatment response criteria for malignant effusion defined in the UK Multi-Centre Study were used to assess the efficacy of this treatment [13]. A complete response (CR) was defined as no re-accumulation of fluid for a minimum of 30 days after treatment, as determined by clinical examination, chest radiography, or echocardiography. A partial response (PR) was defined as minimal fluid re-accumulation, not requiring aspiration, within the initial 30-day evaluation period. Patients requiring re-aspiration within 30 days of treatment were classified as having treatment failure. Patients who died within 30 days of treatment with CBDCA were defined as not evaluated (NE). The evaluation duration ended with either recurrence of effusion or death. Performance status (PS) was evaluated as per the criteria defined by the Eastern Cooperative Oncology Group (ECOG). Clinical characteristics, rate of MPE control 30 days beyond administration, re-accumulation, complications, survival after intrapericardial instillation of CBDCA, time-to-drainage, catheter withdrawal, and adverse events were evaluated based on medical charts.

### 2.3. Statistical Analysis

Survival duration was calculated from the date of initial intrapericardial administration of CBDCA to the date of death or last follow-up. The Kaplan–Meier method was used to estimate the survival time. Adverse events associated with the intrapericardial administration of carboplatin were graded according to the Common Terminology Criteria for Adverse Events version 4.0. All statistical analyses were performed using GraphPad Prism 8 for Windows (SAS Institute, Cary, NC, USA).

## 3. Results

### 3.1. Patient Characteristics

Between January 2005 and March 2018, 21 patients with lung cancer (14 male and seven female), with a median age of 60 years (range: 42–76 years) were administered intrapericardial fixed-dose CBDCA. The patients’ backgrounds are summarized in Table 1. One patient had an ECOG PS of 1, while the PS of the remaining 20 patients was in the range of 2–4. The most common histological type of lung cancer among the patients was adenocarcinoma, which was seen in 13 patients. Three patients were diagnosed with small cell carcinomas. Fifteen patients received prior systemic chemotherapy for the treatment of lung cancer, and six patients were treated with radiotherapy in the thoracic region. The total aspirated effusion volumes ranged from 350 to 11,380 mL (median: 1235 mL). The duration of pericardial drainage ranged from 3 to 85 days (median: 9 days). Of the 21 patients, 18 received one instillation of CBDCA, two received two instillations, and one received three instillations before the drainage tubes were withdrawn. Thirteen patients were discharged after pericardial adhesion.

### 3.2. Treatment Efficacy and Patient Survival

**Figure 1:**
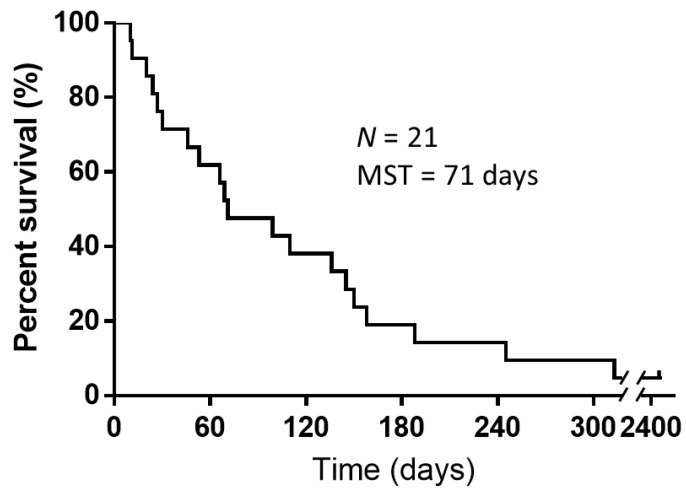
Kaplan–Meier curves for overall survival of patients after intrapericardial instillation of carboplatin. The median survival time from intrapericardial carboplatin administration until death or study follow-up was 71 days (*N* = 21). Abbreviations: N, number of patients included in the study; MST, mean survival time.

Table 2 lists the responses to the intrapericardial administration of 150 mg/body CBDCA. A total of 11 patients achieved CR, and three achieved PR, two patients met the criteria for treatment failure, and five were NE during the follow-up period. The response rate was 66.7% [95% confidence interval (CI): 46.5–86.8%]. The response rate was 72.3% [95% CI: 51.5–92.9%] in the 18 patients who had cancers other than small cell carcinoma. The patients who achieved CR did not experience recurrence after intrapericardial administration of CBDCA until death and had survival times ranging from 31 to 313 days (median 110 days). The five patients who were NE due to the short survival time after treatment (death within 30 days) were not found to have fluid accumulation after treatment, and the death was not attributed to the administration of CBDCA. Of the 14 patients who responded to treatment (CR + PR), 12 were discharged from the hospital, while two could not be discharged. The median follow-up time was 71 days. The median OS time after intrapericardial administration of CBDCA was 71 days (10–2435 days) (Figure 1). By the data cut-off date for this study (31 December 2020), only one patient was still alive.

### 3.3. Feasibility and Toxicity Profiles

Table 3 lists the adverse events observed after the intrapericardial administration of CBDCA. Grade 1–2 pain, nausea, fever, and neutropenia were observed after CBDCA administration. These could be managed by supportive treatment. Therefore, no significant complications were observed, and no patient was found to experience cardiovascular symptoms, such as severe chest pain or arrhythmia after the intrapericardial administration of CBDCA. No treatment-related deaths were documented.

### 3.4. Patient Characteristics and Course of Treatment for Each Patient

Table 4 lists the detailed patient characteristics, treatment outcomes, course of treatment, and prior treatment. Cytotoxic drug therapeutic regimens were the most common forms of chemotherapy administered prior to treatment, and supportive care was the most common post-treatment. Six patients did not receive any treatment, including chemotherapy or thoracic radiotherapy, prior to pericardial adhesion. Four of these patients received systemic chemotherapy after pericardial adhesion, while two did not receive systemic chemotherapy or thoracic radiotherapy. Case 11 was the only driver gene mutation-/translocation-positive patient who was found to be anaplastic lymphoma kinase (ALK) translocation-positive during the course of treatment. The patient was treated with alectinib after pericardial adhesion and survived for 2435 days after the intrapericardial infusion of CBDCA.

## 4. Discussion

Lung cancer is the major cause of MPE, and lung cancer patients with MPE tend to show a particularly poor prognosis. MPE is a potentially fatal complication of cancer. However, the appropriate treatment for intrapericardial adhesion in cancer pericarditis has not yet been determined. A previous review on the treatment of symptomatic carcinomatous pericarditis reported that it is still unclear which treatment modality, i.e., pericardial drainage alone, pericardial adhesion with sclerosing agents, or surgical decompression of the pericardium, is superior [14]. Further and higher-quality studies are needed to resolve the issues raised in that literature review. In this study, we investigated the effectiveness and feasibility of intrapericardial administration of CBDCA (150 mg/body) in patients with lung cancer.

The response rate (CR and PR) to treatment was 66.7%, even though five of 21 patients, including those with small cell carcinoma, were NE and died within 30 days of treatment. The number of NE cases was higher when cases of SCLC with a poor prognosis were included in the current analysis. On the other hand, a response rate of 72.3% was seen when the analysis was limited to patients with NSCLC; this is comparable to the findings of previous studies conducted in patients with NSCLC [8,9,10,11]. In the current analysis, seven patients were treated with systemic chemotherapy, and MPE was regulated; the median survival period from intrapericardial instillation of CBDCA until death or censored was 71 days; this is similar to or slightly shorter than that reported in previous studies (Table 5) [8,9,10,11]. There have been reports of intrapericardial administration of other drugs in various types of cancer pericarditis as well as in lung cancer patients [15,16,17,18]. According to these reports, thiotepa, tetracycline, and cisplatin are capable of controlling 82.6–100% of MPE, while associated with severe complications. Of the 14 patients who underwent pericardial adhesion and responded well, seven received subsequent systemic chemotherapy, and 12 were discharged from the hospital. This may benefit patients who are responsive to pericardial adhesion. Furthermore, because it is not possible to assess whether intrapericardial carboplatin improves survival, preventing the recurrence of malignant pericardial effusions is beneficial because it prevents potentially lethal complications. The median duration of drainage was 9.5–10.5 days in patients with NSCLC, comparable to that described in previous reports [10,11]. Grade 1–2 adverse events were observed, but there were no grade 3 or higher serious adverse events or treatment-related deaths caused by intrapericardial administration of CBDCA. Based on these results, this treatment may be considered effective and feasible, keeping in mind that it was a retrospective study involving patients with a condition that shows a poor prognosis. However, this study included cases from 2005, which was before the introduction of immune checkpoint inhibitors and kinase inhibitors for the treatment of driver gene mutation-/translocation-positive cases. The effectiveness and feasibility of intrapericardial CBDCA in combination with kinase inhibitors and immune checkpoint inhibitors remain unclear and should be further investigated.

Of the 21 patients included in the current study, 14 had CR or PR after pericardial adhesion, and seven received systemic chemotherapy after pericardial adhesion. None of the patients who did not respond to pericardial adhesion or died within 30 days of pericardial adhesion received subsequent systemic chemotherapy. The median survival after pericardial adhesion in the 14 patients who responded to pericardial adhesion was 140.5 days (ranging from 31–2435 days), and the median survival after pericardial adhesion in the seven patients who received systemic chemotherapy was 158 days (ranging from 69–2435 days). Of the seven patients who received subsequent treatment, two received kinase inhibitors and six were given cytotoxic drugs. Six of the cases were adenocarcinoma, while one was large cell carcinoma. Although it is difficult to draw definitive conclusions form the small number of cases analyzed, our data showed that patients responding to pericardial adhesion showed a prolonged survival when they were subsequently administered systemic chemotherapy.

Moriya et al. demonstrated that intrapericardial instillation of CBDCA (300 mg) in patients with NSCLC was effective in controlling MPE in nine of ten patients [11]. Their method involved administrating 300 mg CBDCA and 100 mg lidocaine dissolved in 50 mL normal saline via a tube into the pericardial space. Following instillation of the drugs, the drainage tube was clamped and reopened after 40 min. In our analysis, 150 mg CBDCA dissolved in 20 mL normal saline was administered through a drainage tube, after clamping for 2 h. This dose was chosen keeping in mind the small body size of Japanese patients. Furthermore, we chose a longer clamping duration, since a previous report showed that a concentrated dose of CBDCA in pericardial effusion was effective in killing tumor cells 1.5 h beyond reopening the catheter, and a dilute dose of CBDCA in the plasma allowed the control of MPE with minimal systemic toxicity. This approach helped ensure that the intrapericardial instillation of CBDCA was appropriate for patients who cannot tolerate systemic anticancer agents. We noted no apparent differences in the safety and efficacy between 150 mg and 300 mg CBDCA administered intrapericardially. Therefore, a dose of 150 mg CBDCA may be suitable in clinical practice.

There were several limitations to the current analysis. Firstly, this investigation was a retrospective study; therefore, we could not investigate late cardiac complications, which may introduce bias in the results of our study. Secondly, in contrast to most prospective studies, our exploratory study was not limited to patients who were in good condition or could be expected to show a good prognosis for a certain period. Therefore, the results of this study are likely to more closely reflect the outcomes of clinical practice, since many patients tend to be in a poor condition, such as those with cancerous pericarditis. Thirdly, the current study did not compare intrapericardial instillation of CBDCA with pericardial drainage alone. The cases of carcinomatous pericarditis requiring treatment for pericardial effusion in lung cancer are rare. The low incidence makes it difficult to conduct a prospective trial assessing pericardial adhesion in an adequate number of patients with a single cancerous tumor. Fourth, the period covered was from 2005 to 2018. Although the drainage method was basically the same, the systemic anticancer agents used have changed over time, so it is possible that the course of treatment before and after intrapericardial carboplatin administration affected survival. However, preventing the recurrence of malignant pericardial effusions may be beneficial because it may prevent potentially fatal complications.

## 5. Conclusions

The current study demonstrated that intrapericardial instillation of CBDCA (150 mg/body) is feasible, shows acceptable toxicity, and induces a promising response to lung cancer-associated MPE. However, given the small number of patients and the retrospective nature of the study, further studies are required to explore the effectiveness and feasibility of intrapericardial instillation of CBDCA in patients with lung cancer-associated MPE.

## Figures and Tables

**Table 1:** Patient characteristics.

Characteristic	Number of Patients (*N* = 21)
Gender	
(male/female)	14/7
Median age at drainage (years)	60 (42–76)
Performance Status	
(0/1/2/3/4)	0/1/7/12/1
Histology	
(Adenocarcinoma/squamous cell carcinoma/small cell carcinoma/others)	13/4/3/1
Driver gene mutation/translocation	
(Yes/no or unknown)	1/20
Prior chemotherapy	
(Yes/No)	15/6
Post chemotherapy	
(Yes/No)	7/14
Prior thoracic radiotherapy	
(Yes/No)	6/15
Drainage methods	
(Catheter/others)	21/0
Median drainage volume (mL)	1235 (350–11,380)
Effusion cytology	
(Positive/negative/unknown)	21/0/0
Duration of drainage (days)	9 (3–85)
Number of times carboplatin was administered	
(1/2/≥3)	18/2/1
Discharged from hospital	
(Yes/No)	13/8

**Table 2:** Response evaluation.

Treatment Response	Number of Patients (*N* = 21)	Patients (%)	Number of Patients Excluding Those with SCLC ^a^ (*N* = 18)	Patients Excluding Those with SCLC ^a^ (%)
Not evaluated	5	23.8	3	16.7
Evaluated	16	-	15	-
Complete response	11	52.4	10	55.6
Partial response	3	14.3	3	16.7
Treatment failure	2	9.5	2	11

^a^ SCLC, small cell lung cancer.

**Table 3:** Incidence of treatment-associated adverse events.

Adverse Events	Grade 1	Grade 2	Grade 3	Grade 4
Pain	3	0	0	0
Nausea	2	0	0	0
Fever	2	0	0	0
Neutropenia	1	1	0	0

**Table 4:** Patient characteristics, course of treatment, and treatment outcomes.

Case	Age	Sex	PS	Histology	Prior Therapy	Post Therapy	Volume of Effusion (mL)	Duration of Drainage (Days)	Number of Intrapericardial Doses of CBDCA Administered	Discharge from Hospital	Response	Survival after Intrapericardial Infusion (Days)
1	76	M	4	SQ	None	None	1235	15	1	No	NE	10
2	64	M	3	SCLC	CBDCA + VP-16 → CBDCA + CPT-11	None	3510	15	2	No	NE	11
3	69	M	3	Large	None	VNR	1720	10	1	Yes	CR	150
4	55	F	2	AD	CDDP + GEM → CBDCA + PTX → DTX → GEF	GEF	550	8	1	Yes	CR	110
5	64	M	3	AD	None	CBDCA + PTX → DTX → GEF	900	7	1	Yes	CR	313
6	48	F	3	AD	DTX + GEM → GEF	None	350	4	1	Yes	PR	188
7	70	M	3	AD	CBDCA + PTX→ DTX + S1	None	1200	3	1	Yes	Failure	46
8	42	M	2	SCLC	CBDCA + VP-16 + TRT → CBDCA + CPT-11	None	860	8	2	No	CR	31
9	63	M	2	SCLC	CBDCA + VP-16 + TRT → AMR → CBDCA + CPT-11	None	1400	4	1	No	NE	20
10	63	M	3	AD	CBDCA + PTX → DTX	None	450	5	1	Yes	CR	145
11	67	F	2	AD	CDDP + S1 + Bev → TRT → CRZ	PEM → ALC	540	3	1	Yes	PR	2435 ^a^
12	51	M	3	SQ	CBDCA + PTX + TRT → DTX → S1 → ERL → GEM	None	3160	7	1	Yes	CR	53
13	50	F	3	AD	CBDCA + DTX + Bev → PEM → ERL	None	640	4	1	Yes	CR	99
14	44	M	2	AD	CBDCA + PTX + TRT → CDDP + VNR	None	11,380	85	3	No	Failure	66
15	59	F	3	AD	None	CBDCA + PTX	450	10	1	Yes	CR	69
16	59	F	3	AD	CBDCA + DTX + Bev	DTX	1285	10	1	Yes	CR	158
17	60	F	2	AD	None	CBDCA + PTX → PEM	1450	9	1	No	CR	71
18	58	M	3	AD	CDDP + S1 → DTX	None	1555	9	1	No	NE	24
19	46	M	2	AD	None	None	1270	14	1	No	NE	27
20	67	M	1	SQ	CBDCA + PTX + TRT	None	880	9	1	Yes	PR	136
21	69	M	3	SQ	CBDCA + PTX	None	2050	11	1	Yes	CR	245

^a^ Alive at cut-off date. Abbreviations: PS, performance status; SQ, squamous cell carcinoma; SCLC, small cell lung cancer; Large, large cell carcinoma; AD, adenocarcinoma; CBDCA, carboplatin; VP-16, etoposide; CPT-11, irinotecan; CDDP, cisplatin; GEM, gemcitabine; PTX, paclitaxel; DTX, docetaxel; GEF, gefitinib; TRT, thoracic radiotherapy; AMR, amrubicin; Bev, bevacizumab; CRZ, crizotinib; ERL, erlotinib; PEM, pemetrexed; VNR, vinorelbine; ALC, alectinib; NE, not evaluated; CR, complete response; PR, partial response.

**Table 5:** Pericardial sclerosis for the management of malignant pericardial effusion in patients with lung cancer [8,9,10,11].

Agent	Dose Administered	Patient Number	Histology (NSCLC/SCLC ^a^)	Number of Cases Successfully Controlled/Total Number of Patients (%)	Adverse Events	Median Survival (Days)	References
Carboplatin	300 mg/body	10	10/0	8/10 (80%)	None	69	Moriya et al. (2000) [11]
Mitomycin C	2 mg/body	8	8/0	6/8 (75%)	None	80	Kaira et al. (2005) [10]
Bleomycin	10 mg/body	22	22/0	21/22 (95%)	Anemia (*n* = 2), Hypoalbuminemia (*n* = 3), Liver dysfunction (*n* = 3), Hyponatremia (*n* = 2), Fever (*n* = 6), Sinus tachycardia (*n* = 2), Leukopenia (*n* = 2)	125	Maruyama et al. (2007) [9]
Bleomycin	15 mg/body	38	36/2	25/38 (65%)	Fever (*n* = 2), Pain (*n* = 13), Infection (*n* = 3), Bleeding (*n* = 2), Cardiac dysfunction (*n* = 1), Constrictive pericarditis (*n* = 1)	119	Kunitoh et al. (2009) [8]
Carboplatin	150 mg/body	21	18/3	14/21 (67%)	Pain (*n* = 3), Nausea (*n* = 2), Fever (*n* = 2), Neutropenia (*n* = 2)	71	Current study

^a^ NSCLC, non-small cell lung cancer; SCLC, small cell lung cancer.

## Data Availability

The raw data from this study are available from the corresponding author upon reasonable request.
